# Examining barriers and facilitators of dental fear treatment adoption: A qualitative study of practicing dentists

**DOI:** 10.1371/journal.pone.0322884

**Published:** 2025-05-09

**Authors:** Jennie Ochshorn, Kelly A. Daly, ViniNatalie Zaninovic, Richard E. Heyman, Amy M. Smith Slep, Mark S. Wolff

**Affiliations:** 1 New York University College of Dentistry, New York, New York, United States of America; 2 University of Pennsylvania School of Dental Medicine, Philadelphia, Pennsylvania, United States of America; Danube Private University, AUSTRIA

## Abstract

Over fifteen percent of the global population experiences dental fear, and although evidence-based treatments exist, adoption of these treatments is almost non-existent. Informed by our prior research examining barriers to adopting face-to-face behavioral treatments in dental operatories, this study examined dentists’ responses to three stepped-care Cognitive Behavioral Therapy for Dental Fear (CBT-DF) formats that use technology. All approaches offer an automated component as the first step (a mobile app) and either an in-person, virtual reality (VR), or video telehealth session as the second step. This study aims to understand which of these approaches would most likely be adopted by private practice dentists and why. Eight focus groups/solo interviews with a total of 13 private practice dentists were conducted with the aim to assess barriers and facilitators to implementing three stepped-care approaches of CBT-DF. The qualitative data obtained from these interviews was coded and analyzed according to Rogers’ framework of innovation (relative advantage, compatibility, complexity, trialability, and observability). The results indicated that participants acknowledged the value of interventions to address dental fear, as they had personal experiences with fearful patients that impacted their practices. Participants responded positively to the automated component of treatment (the app) and were more wary of treatment options requiring office space and staff time (in-person VR and in-person mental health provider). The telehealth option received the most favorable response, although some doubts were expressed regarding relative efficacy and patient accountability. Thus, dissemination of an app-telehealth treatment model that allows dentists to serve as referral partners is promising, given dentists’ incentives to decrease patient fear while avoiding opportunity cost (e.g., occupied chairs and staff time).

## Introduction

The dissemination and implementation of evidence-based interventions (EBIs) is a global public health priority [1]. The sluggish pace of adoption of EBIs across health policy and practice has served as the primary catalyst for the emergence and development of an entire discipline, implementation science [2]. Implementation science focuses on understanding the barriers and facilitators to the adoption and integration of scientific findings and evidence-based practices across systems [3] and provides a broad framework for contextualizing the phenomenon of dental fear, a major public health concern [4].

The prevalence of clinically significant dental fear among adults is 15.3% globally [5]. Among fearful individuals, anticipatory distress about dental visits often leads to avoidance of routine care which, over time, compromises oral health and increases the need for complicated and expensive dental procedures [5]. Dental fear is widespread and consequential, resulting in needless pain and suffering, oral and systemic health decline, and financial and social costs [6]. However, dental fear is also highly treatable; Cognitive Behavioral Therapy for Dental Fear (CBT-DF) has demonstrated efficacy across two dozen clinical trials with large effect sizes [7,8]. Yet, this highly effective treatment remains largely inaccessible to the millions of adults who could benefit from it [9]. Indeed, CBT-DF is rarely offered outside of a few specialty clinics worldwide, few mental health providers are trained in it, and dental practitioners are largely unaware of its existence [10].

Implementation science has been criticized for over-relying on researchers’ perceptions of healthcare practice rather than eliciting perspectives of actual full-time providers [11]. Thus, we have collaborated extensively with dentists and allied professionals to assess barriers to incorporating CBT-DF treatment. A previous study assessed responses from a focus group of dentists about providing CBT for dental fear via an evidence-based collaborative care model (i.e., embedding a mental health provider within a dental setting to provide psychological services when appropriate [9]). Although dentists were generally receptive to the concept of CBT-DF treatment, they expressed significant concerns regarding the financial and practical feasibility of using traditional evidence-based collaborative care [12] in dental settings [9]. Notably, traditional CBT-DF requires about three hours to administer in a dental chair within private practice or clinic settings [8]. We encountered hesitancy amongst dental providers regarding collaboration with mental health professionals directly in the dental home—especially if collaboration meant ceding significant chair time (otherwise available for dental procedures) and space (potentially disrupting workflow) to accommodate a mental health professional. Dentists cited the resulting loss of income as a significant concern [9]. It should be noted that although dentists may see this financial burden as a limitation, like other medical professionals, they have a responsibility to uphold ethical standards. Additionally, dentists questioned the feasibility of an in-chair intervention for their most fearful patients, noting the difficulties they have getting these patients to attend any dental visits [9].

Due to the COVID-19 pandemic, telehealth treatment options have become widespread, offering the possibility of innovative psychological treatment models that reduce logistical and cost barriers [13–15]. Moreover, a precedent existed for the development of technology-facilitated dental fear interventions. The dental fear treatment using computerized CBT [16], as well as mobile tools delivering exposure therapy [17], have been piloted by others with promising results. Informed by earlier research in this domain, we developed three different stepped-care (i.e., adaptive dosing) models of CBT-DF that we felt might address prior logistical and cost barriers identified by dental professionals.

Stepped-care intervention prioritizes parsimony, providing individuals with an initial low dose of treatment; if the patient does not benefit adequately [18–20], more intensive (or higher dose) interventions are administered. All three stepped-care models use technology to mitigate logistical and cost barriers and share the same first step (a self-administered CBT-DF mobile app accessed from smartphones at the participant’s convenience [21]). We believed that automating the foundational, lower-dose level would allow for significant reductions in costs (i.e., removing the scheduling complications and expense of a mental health provider, not requiring chair time) and increased feasibility (i.e., 24-hour availability whenever convenient for the patient, no disruption of practice workflow, accessibility to patients without current dental providers). The second step varied across models: (1) in-operatory treatment by a mental health provider, (2) remotely administered telehealth session with a mental health provider (eliminating practices’ space/cost concerns and allowing dentists to refer fearful patients externally), and (3) in-person virtual reality (VR) to administer the CBT-DF via immersive technology that eliminates the mental health provider (and obviates the need for practices to have a non-dental provider in their operatories).

Our previous work revealed dentists’ considerable concerns that cost, time, and logistics were barriers to welcoming comprehensive in-person, evidence-based psychological treatment in dental practices. In this paper, we updated the proposed CBT-DF approaches (i.e., technology-assisted) and used the Diffusion of Innovation Theory [22] as a framework for analyzing dentists’ reactions to the three proposed stepped-care models. Rogers et al. (1995) theorized that understanding how potential adopters perceive barriers and facilitators of an innovation can shed light on future disseminability. Based on the existing research, we hypothesize that most dentists will opt for the entirely virtual intervention that provides the least logistical barriers for their practice.

## Methods

### Participants and procedures

Participants were dentists in private practice (*N *= 13). This self-selected sample comprises dentists who volunteered to participate in an uncompensated Zoom focus group/interview. We solicited NYU-affiliated private-practice practitioners, including alumni, via email. Before participating in the focus groups/interviews, dentists signed written consent to participate in the study (NYU IRB#: IRB-FY2022–5951) and completed a demographic questionnaire that included questions related to professional dental experience (see Table 1). The recruitment period for this study began on 12/10/21 and ended on 5/10/22. Eight separate focus groups/interviews, ranging from 1 to 4 participants, were conducted to gauge the potential benefits and barriers of integrating each of our stepped-care approaches in private practice settings. All interview transcripts from the Zoom recordings were de-identified.

**Table 1:** Demographic characteristics and dental-related questions (N = 13).

Variable		*M* (*SD*) or *n* (%)
Age (years)		48.77 (15.47)
Gender	Female	11 (84.6%)
	Male	2 (15.4%)
Race	Native American or Alaska Native	0 (0%)
	Asian	1 (7.7%)
	Native Hawaiian or Other Pacific Islander	0 (0%)
	Black or African American	2 (15.4%)
	White	10 (76.9%)
	More than one race	0 (0%)
	Other	0 (0%)
	Don’t know	0 (0%)
Ethnicity	Hispanic	1 (7.7%)
	Non-Hispanic	11 (84.6%)
	Other	1 (7.7%)
Do you currently practice in a private dental office?	Yes	8 (61.5%)
	No	4 (30.8%)
	Missing	1 (7.7%)
Throughout your career, approximately what percentage of your patients have had moderate to severe dental fear?	10% or less	6 (46.2%)
	11 - 30%	2 (15.4%)
	31 - 50%	2 (15.4%)
	51 - 70%	2 (15.4%)
	71% and above	1 (7.7%)

Each focus group/interview took place on Zoom and began with a description of the evidence-based CBT intervention we have adapted and an explanation of our stepped-care approach — an interactive mobile app plus either (a) an in-person one-on-one session with a mental health provider, (b) a telehealth one-on-one session with a fear specialist or (c) a virtual-reality device with custom dental fear treatment software. Participants were given a detailed description of each model as outlined in Table 2 and given opportunities throughout the interviews to ask clarifying questions. These focus groups/interviews were facilitated by either a clinical psychologist or a dental hygienist from our research team. All facilitators were trained, observed, and given a question guide to ensure accuracy and consistency.

**Table 2:** Intervention models and descriptions.

Step 1		
Inter-vention	Content	Logistics
CBT-based Mobile App	Interactive/educational modules Psychoeducation topics cover common fears experienced and cognitive behavioral strategies for coping at the dentist Modules include 1) painful and uncomfortable procedures, 2) communicating with the dental team, 3) managing bodily symptoms, and 4) needles and injections Throughout app modules, participants learn to modify what they feel, think, and do at dental visits	App can be done from a tablet or smartphone Takes about an hour to complete Completed prior to dental appointments Is entirely virtual and self-administered
Step 2		
In-Chair One-On-One Treatment	CBT session combines exposure and other CBT techniques Patient practices principles learned in app (i.e., managing what they feel, think, and do) Feared situations simulated in the dental chair with real dental tools After practicing feared situations, the provider and participant debrief, focusing on fear disconfirmation	In-person one-on-one intervention at a dental office in a dental chair Exposure session with mental health provider Takes about 1 hour to complete
Telehealth One-On-One	CBT session combines exposure and other CBT techniques Patient practices principles learned in app (i.e., managing what they feel, think, and do) Feared situations simulated over Zoom After practicing feared situations, provider and participant debrief, focusing on fear disconfirmation	Telehealth one-on-one intervention conducted over Zoom Exposure session administered by a mental health providers Takes about 1 hour to complete
Virtual Reality	VR session combines exposure and other CBT techniques VR dentist simulates an exam and restoration; coach provides assistance during exposure Patient practices principles learned in app (i.e., what patient feels, thinks, and does) Disconfirmation occurs at next dental visit	VR device administered in a dental chair Staff member gets patient set up Takes about 1 hour to complete

Participants were asked about their experiences with fearful patients and their biggest issues in addressing dental fear. They were then asked about the challenges and benefits of each intervention, how it could be integrated into their practice, and how it compares to what they currently have in place. Toward the end of each discussion, participants were asked which of the three treatment approaches would be the best fit for their office. The average length of the interviews was approximately 40 minutes. Participants were interviewed until thematic saturation was obtained. For each discussion prompt, the facilitator ensured that each participant was given the opportunity to engage in conversation before moving on to the next prompt. Descriptions of the intervention models (Table 2) are provided below.

### Data analysis

We conducted a multi-step thematic analysis using Braun and Clarke’s (2006) codebook thematic analysis approach [23] to qualitatively analyze the main themes from each focus group/interview. Our coders highlighted the key excerpts from each transcript and reduced each quote to its underlying theme. Two other researchers independently analyzed and grouped these themes according to a codebook we developed based on Rogers’ five attributes for predicting the rate of an innovation’s adoption/diffusion (relative advantage, compatibility, complexity, trialability, and observability [24]) along with their counterparts (disadvantage, incompatibility, usability, and lack of trialability/observability). Definitions of these drivers (Table 3) are provided below. Disagreements in coding were resolved via consultation and discussion among the full team.

**Table 3:** Definitions of drivers and within context.

Drivers	Definitions [24]	In Context of Current Study
Relative Advantage	“The degree to which an innovation is perceived as being better than the idea it supersedes […] often expressed as economic profitability, social prestige, or other benefits.”	The perceived effect of the intervention on dental practices. Often involving factors such as cost, time, and space. Will the intervention be easily integrated into the office? Is this an improvement to what the dental practice currently has in place? Or does it create an inconvenience for staff?
Compatibility	“The degree to which an innovation is perceived as consistent with the existing values, past experiences, and needs of potential adopters.”	In this case, “adopters” refers to the patients who will be using the intervention. The patient’s needs/values must be compatible with the technology. Will they be interested in the content? Is there a need for it? Will there be time or cost limitations on the patient’s end?
Complexity	“Complexity is the degree to which an innovation is perceived as relatively difficult to understand and use.”	How easy to use is the innovation? Is it accessible to disadvantaged groups?
Trialability	“Trialability is the degree to which an innovation may be experimented with on a limited basis.”	Can practices try out the innovation before implementing it? Can they review the software/content before deciding whether to implement it?
Observability	“Observability is the degree to which the results of an innovation are visible to others.”	Have similar interventions been used in practice before? If so, what did we learn? What evidence can be provided to show the effectiveness of the innovation?

## Results

Themes were grouped according to Rogers’ five major attributes for predicting the rate of adoption [24], along with their supporting quotations. Expanded exemplars are presented in Table 4.

**Table 4:** Grouping codes according to drivers and themes.

Disseminability Drivers		Themes	Quotes
App			
*Relative advantage* over current practice	Advantage	Can be completed before appointment	“I think that if we can get them to do it before they come in, that’s feasible. But if they’re already there for their appointment, like you’ve missed the mark.”
			“I think that’d be very doable to have something you email to your patient before they come in or send to the patient an hour before so that they can work on it before [their appointment].”
		Screening capabilities	“It’s really good if we can find out who has real dental fear and not just, you know, discomfort.”
			“Trying to get to the root of the problem before the appointment […] I could pull up their chart even before we do anything, I think that would be helpful.”
		Won’t disrupt practice	“You know, pre-appointment, I think it’s fine because it doesn’t impose on any kind of time in the office. […] That’s done at home on their own time and nothing you’ve described right now has to do with putting the patient in the chair.”
	Disadvantage	Can already evaluate/treat patient fear	“I basically try to assess from a patient whether they have had negative experiences and if they are fearful. I’ve taught some patients yoga breathing because I do yoga and meditation myself.”
		Not time/cost efficient for practice	“I may not directly ask them, but I try to figure out what’s going on with them and make them feel comfortable […] We try to recognize it at the beginning and deal with it.”
*Compatibility* with prevailing ideals, attitudes, and experience	Compatibility	Questionnaires increase patient ease	“She [the patient] said she filled out the fear assessment and just going through that made her very calm. You know, the fear assessment explained, if you have questions, if you’re in pain, we’ll notify the dentist […] She said that alone quelled her fear.”
		Anonymity	“Sometimes you ask something and, like I say before, some people feel like oh, I don’t want to answer this question why everybody needs to know about me.”
			“It’s totally, like judgment free […] potentially their first time either putting it into words or verbalizing or admitting to themselves […] seeking some more understanding or, you know, kind of treatment without having to go directly to, you know, a professional […] just kind of like exploring the topic themselves.”
		Patients with serious fear will be motivated (to complete app)	“If it’s someone who is truly affected by fear and really does struggle to come into the office […] those patients really should, and I think would kind of take the time to actually, you know, utilize it [the app].”
	Incompatibility	Not needed (for patients)	“Most of the patients that do come into the office, some of them do need treatment, but most of them are like our reoccurring patients that really don’t need that much.”
		Patients don’t have time	“The only negative I see is that if it takes an hour to an hour and a half to have a patient go through it, that might be a negative because I treat a lot of high-powered executives and people in Manhattan as you know, are pressed for time already.”
*Complexity*: complication with using innovation	Usability	Apps are accessible	“I think people like to use apps. They like to work on their cell phones.”
		Apps are engaging	“If it’s something interactive, I feel like it’s something I can promote to them as exciting and helpful. That it’s like an exercise and not just information. Sometimes patients are like, ‘Oh, it’s just more information,’ they don’t really want to take the time to do it.”
	Complexity	Accessibility for older/disabled patients	“Some of the older people aren’t very tech savvy, and using an app is not something that would be easy for them to do.”
			“If you have a patient with disabilities, he’s going to need someone else to explain you know, the question, and then the subsequent questions that go with it.”
*Trialability*: ability to study innovation	Trialability	N/A	
	Lack of trialability		
*Observability* of both the innovation and its impact	Observability	N/A	
	Lack of Observability	Needs evidence of effectiveness	“You’d want to have evidence that when people become less fearful, they become more compliant. Showing that data […] would probably encourage them [dentists] to implement this [the app] with their fearful patients.”
In-Chair One-on-One			
*Relative advantage* over current practice	Advantage	Replace/reduce medications	“We can mark it as non-medication based, you know, anxiety therapy. Which I think a lot of people, especially nowadays, are trying to take less and less medications.”
			“Just giving the patient the option, like, ‘Hey, if you try this and you’re still afraid, we can still offer you medications.’”
		Flexibility	“It would probably be feasible if it was not busy times. Like late mornings sometimes tend to not be as busy […] Weekends are challenging […] So I think it would be feasible, it just has to be a designated time.”
			“I think it may work for my office because I have two separate rooms with closed doors, and we will all only see one patient at a time.”
		Increase financial gains (if patients resolve fear)	“It may increase income on the backend because you’ll have a more compliant patient who’s going to keep their appointments, get the care they need, and hopefully complete a treatment plan that maybe they wouldn’t have without the intervention.”
			“I have had patients that were so fearful that I either couldn’t continue treatment or like, oh, my gosh, I just want this patient out of my office because I just can’t do it. And it wastes so much time.”
	Disadvantage	Time/cost inconvenience	“Probably like not even possible, because we have like limited chairs and extremely high volume.”
			“Our office is the busiest office […] we don’t have an empty chair at all.”
		Can already evaluate/treat patient fear	“I’m pretty well-skilled at this point in keeping people calm. […] I mean, I’m able to work with people pretty well to get to the core of their anxieties and fears.”
*Compatibility* with prevailing ideals, attitudes, and experience	Compatibility	Effectiveness	“I think the most effective would be in the chair with the person for sure, because that’s the location where it’s happening.”
			“I understand the importance of sitting in the chair and going through the motion and desensitization.”
	Incompatibility	Time inconvenience	“Do they have to take time off work or [use] their weekend hours? Like childcare, like all that stuff, is really going to be a barrier for them […] and you know, who’s going to drive them and stuff like that.”
			“I don’t think it will work for my office, because people are at a very fast-paced in the city […] they have their hour, 45 minutes for the appointment. And they just want to go.”
		Not needed	“I don’t know that any of my patients would necessarily need that [the intervention]. And I don’t have patients in my practice whom I would say are severely anxious as to need these one-on-one sessions.”
			“We assume everybody who is coming, they come in because they have to, not because they want to. And we kind of like, you know, get used to it.”
		Lack of privacy	“Ortho[pedic] office setups are very different than dental. It’s like six chairs, but they’re all open to each other. So, you couldn’t have a dental fear specialist with one patient. It wouldn’t be private. We only have one private room.”
			“I don’t think anybody would feel comfortable if that whole experience is happening when someone is coming out of a chair almost directly next to them, which is how it is there.”
		Potential stigma/embarrassment	“Is there an issue with having a little embarrassment or stigma of like, you know, they’re afraid of the dentist or something like this. But doing it in on a day, say, that no one else is in the office and it’s just the specialist and the patient [would be feasible]. “
*Complexity*: complication with using innovation	Usability	N/A	
	Complexity		
*Trialability*: ability to study innovation	Trialability	N/A	
	Lack of Trialability		
*Observability* of both the innovation and its impact	Observability	N/A	
	Lack of Observability	Needs success rate	“I was just curious to know the success rate once they actually went through the therapy session. How much better do they feel, and do they actually become a more compliant patient instead of postponing their dental work?”
			“When you’re starting something new, and no one knows if it’s gonna work, you’re not willing to make the sacrifice with the time. But if you feel that it’s going to work, and it’s a proven method, then you should sacrifice the time because in the long run, you’re gonna make that money.”
		Needs details of treatment/financial aspects	“Is that dental fear specialist coming in and paying you to use your chair? Are you paying them?”
			“I guess the patient would have to what the fee would be. And perhaps they want to know what the qualifications of the facilitator were.”
Telehealth One-On-One			
*Relative advantage* over current practice	Advantage	More feasible (than in-chair)	“It’s a much better idea, a much better solution, to do it via Zoom, as opposed to occupying an office space for an hour.”
			“I don’t have a problem with them doing it virtually, I really don’t. That sounds better than in the office.”
		Replace/reduce medications	“If you do have dental fear, and you want to try a non-medication-based approach, take this questionnaire. […] if you try this and you’re still afraid, we can still offer you medications, we can still put you to sleep for it, if that’s what you want to do.”
	Disadvantage	N/A	
*Compatibility* with prevailing ideals, attitudes, and experience	Compatibility	Convenience	“To be able to do something like this in the comfort of your own living room, as opposed to having to schlep into the dental operatory and do it there, is probably less stressful for the patient.”
			I like it better. It’s more convenient for the patient.
	Incompatibility	Less effective (than in-chair)	“It’s not fully immersive, so you don’t hear the noises, and you don’t smell our dental smell.”
			“It’s certainly easier to do it virtually. But I don’t know, necessarily that will be as effective.”
		Not needed	“I can usually manage most of my patients’ anxieties and fears with my present toolbox.”
		Less patient accountability	“You’ll be waiting on the call and waiting […] Like, ‘oh, I forgot. I forgot to set my alarm,’ or whatever little excuse that they come up with.”
			“Having it be, you know, on their own time can definitely have some challenges as far as actually showing up, if it’s that easy to kind of flake away from it.”
*Complexity*: complication with using innovation	Usability	N/A	
	Complexity	Information barrier	“If you explain it to them correctly, which, you know, would be on staff and myself to do that, I think that people would be receptive as long as we gave them all the information.”
*Trialability*: ability to study innovation	Trialability	Request mock session	“I would want to have almost like a mock session with the behavior specialist so it [the intervention] can be explained. […] To understand what they’re going to be telling them [the patients], see what the approach is, and see like, hey, will this work for our patient pool?”
	Lack of Trialability		
*Observability* of both the innovation and its impact	Observability	N/A	
	Lack of observability	Needs success rate	“Success rates would be something really nice for patients to know. If they have dental fear and I recommend them to go see dental fear specialists, I want them to have some confidence of the likelihood of it actually benefiting them.”
		Needs to gauge patient interest	“What is the amount of patients that would sign on to it?“
Virtual Reality			
*Relative advantage* over current practice	Advantage	Cost benefits	“If you’ve got true success using it to overcome the fear. If you get enough test subjects to be able to say it’s worth your purchase. Because they’re only what, a couple 100 bucks for VR headset, programs, and everything […] I think that would be a fabulous alternative.”
	Disadvantage	Time/Cost Inconvenience	“Having to manage chair time with a patient, having this kind of modality, is more work for my staff.”
			“Sometimes, it’s hard enough for me to get like appliances I need and things for my dental equipment. So, to get something additional, like VR equipment for dental fears, that’s kind of a big expense.”
			“To invest money into these patients who historically don’t come to the dentist, who you know, don’t want a lot of treatment, you have to think of it the business aspect of it.”
*Compatibility* with prevailing ideals, attitudes, and experience	Compatibility	VR is popular now	“Virtual reality is good, especially for young people. Because everybody likes gadgets nowadays, right?”
			“I think our patient pool would be ideal for the VR.”
			“Seems kind of like a next-level option that would be awesome to have and should be incorporated.”
	Incompatibility	Not needed	“I don’t have the patient population who is necessarily in need of this kind of stuff.”
		Missing human connection	“We’re social people, we just prefer to look eye to eye. […] [VR] might look cool, but it’s still not the human being.”
			“I think it’s talking to a human being- it’s, it’s still better.”
		Time inconvenience	“I guess the virtual would be best because, you know, they do it on their own time. Again, it’s a time issue for them.”
*Complexity*: complication with using innovation	Usability	N/A	
	Complexity	N/A	
*Trialability*: ability to study innovation	Trialability	N/A	
	Lack of Trialability	Needs details of experience	“Just to have exactly what the script is, right, exactly what you would be doing.”
			“I think also just making sure everyone’s trained on it properly. And everyone knows exactly what to expect before and after.”
			“Are they [patients] going to experience it? As a patient sitting in the chair? Or is it just like watching a video of someone else going through it?”
*Observability* of both the innovation and its impact	Observability	N/A	
	Lack of Observability	Needs success rate	“Let me have other people do it first, see if it works, and then I’ll jump on board and do it.”
			“I think that one would probably need like good data. I don’t know how many people would want to be like the guinea pig on that.”

### Dentists’ exposure to patient dental fear

Most dentists in the focus groups/interviews reported encountering patients who exhibited fear during their dental visits. Dentists believed fear often leads patients to avoid follow-up appointments, neglect their oral health, or opt for sedation and medication the dentists perceive as unnecessary. Some of the dentists discussed their experiences with patients whose fear stems from bad childhood experiences with dental work. One participant noted, “Most of our people who are really scared of dentists have been traumatized as a child.” Another dentist commented, “That childhood bad experience and that trauma can really stick with you.” It was also mentioned that parents can contribute to dental fear by modeling anxiety to their children. Dentists described different manifestations of fear they have witnessed, such as patients crying or shaking. One dentist described a patient who “is reluctant to open her mouth, and she just, she cries […]. She wants to try, she wants to, but for some reason, she just is very, very jittery.” Another dentist commented, “I have had patients who were so fearful that I either couldn’t continue treatment or like, oh, my gosh, I just want this patient out of my office because I just can’t do it. And it wastes so much time.” Only a few participants reported not having many experiences with fearful patients. One participant remarked, “I cannot say [I see] a lot of patients who have dental fear, but maybe they just hide it.” In addition, some dentists felt that the recent COVID-19 pandemic contributed to anxiety, both because of the need to remove masks at appointments and a general atmosphere of fear around transmission of viruses. Across focus groups/interviews, dentists expressed the ongoing difficulty of convincing fearful patients to return and receive necessary treatment, especially after an introductory consultation or initial exam.

### Step 1: The App

The first step across the presented stepped-care models is *Dental Fearless*, a CBT-based mobile app containing psychoeducation about anxiety and dental procedures and evidence-based CBT strategies and skills [25]. The app consists of 5 modules (participants complete the introductory module and 1–4 of the topic-specific modules), each including psychoeducation, modeling, and practice. Throughout the app, users create personalized plans for managing their anxiety. Typically, the app takes 1–1.5 hours to complete.

#### Challenges.

Dentists in the focus groups/interviews discussed potential barriers to patients using the app in their practices. Identified factors primarily fell under Rogers’ (2003) *relative disadvantage*, *incompatibility complexity*, *lack of trialability*, and *lack of observability*. *Relative disadvantage* was repeatedly evoked across groups by the burden dentists assumed would be placed on staff to instruct patients in downloading and using the app. This burden is a time/cost concern, as it requires the attention of staff during working hours. In addition, dentists emphasized perceived *incompatibilities* on the patient’s end, specifically that patients may not have time to use the app. Participants agreed that their patients have busy schedules and felt that getting them to pursue the app on their own time may be difficult. Furthermore, participants discussed aspects of *complexity* when implementing this technology. The issue of accessibility came up multiple times, specifically concerning older or disabled patients. Some dentists fear this will place yet another burden on their staff if it becomes necessary to spend extra time training patients who are not familiar with using apps or have difficulty navigating the content. General skepticism toward the app was evident, demonstrating a *lack of observability.* Some dentists felt that presenting data on the efficacy of this intervention would increase the likelihood of implementation in practices.

#### Benefits.

Despite the previous shortcomings, participants overall agreed that the app is a convenient and useful intervention, particularly since it can be done outside of the dental office. Positive insights fell under Rogers’ *relative advantage*, *compatibility*, *usability*, *trialability*, and *observability*. The main *relative advantage* discussed by participants is that the app would not disrupt practice workflow, as it can be completed before appointments; this would also reduce likelihood of staff burden to facilitate it. Additionally, dentists felt the app data could have clinical utility for them; they discussed employing it as a screening tool for their offices to identify fearful patients (if permitted access to patient app data). Most dentists reported not having a standard fear screening method in place and thought the app could be a useful remedy. Many participants thought it would be easy to add a link to the app on their websites or integrate access into appointment scheduling processes. Participants also appreciated the possibility of separating severely fearful patients from moderately fearful ones using the app data.

Multiple dentists also mentioned anonymity as an aspect of *compatibility* on the patient’s end. Dentists responded positively to the privacy that accompanies the app versus assessing fear in person or via phone, citing that the app may encourage more honest answers and actual engagement with a fear intervention. Finally, participants emphasized the accessibility and engaging nature of this technology, coded under the *usability* attribute. Dentists agreed that apps are widely used, and most patients will likely be familiar and comfortable with the technology. Participants also commented on the interactive nature of the app, which they felt would garner engagement from their patients. One dentist remarked that the app would be far more effective than giving patients an informational pamphlet to read about dental fear.

### Step 2: In-chair

The first intensive treatment option follows completion of the app and is conducted in a single, one-hour session in a dental chair with a trained mental health provider. This treatment relies on exposure techniques and other CBT techniques and is individualized to a patient’s specific fears.

#### Challenges.

Dentists identified many challenges with the in-person intervention. Concerns were expressed over financial feasibility, COVID considerations, organizational needs, time constraints, and limited chair availability. Participants expressed a concern over the time/cost inconvenience created by in-person sessions; this *relative disadvantage* was a recurring theme throughout the discussion of in-chair treatment. Identified challenges included finding space and time for psychological treatment, as most dentists agreed there are hardly ever available chairs during office hours. Furthermore, dentists felt that the pandemic had increased difficulties with patient attendance at regular in-person appointments, let alone for extra treatments. Some participants felt that while it may be possible to accommodate an in-chair intervention, it would be difficult and time-consuming. A minority of dentists voiced that they can already evaluate and treat fearful patients independently and thus have no need for this intervention. This sentiment was far less endorsed than logistical obstacles.

The main *incompatibility* discussed was patients’ time constraints. This theme recurred frequently, as an in-person intervention requires more effort on the patient’s end than a remote option would. Many participants discussed the difficulty their patients have in finding the time to come in person for basic oral healthcare appointments, which often require finding childcare or taking time off work. Again a few dentists felt the intervention was irrelevant to their clientele. One participant commented that even their fearful patients could come in and “tough it out” during their procedures. A further *incompatibilit*y was the embarrassment dentists worried their patients might feel in seeing a mental health specialist in a (more public) dental setting. This lack of privacy applied to dentists with practices that had open layout floorplans and did not have the spaces behind closed doors to host these sessions. For an in-person treatment especially, participants emphasized that *lack of observability* creates a barrier to implementation. Dentists reported wanting to know the intervention’s success rates and value before considering integrating such a treatment. Additionally, participants felt they would need more compelling evidence regarding the necessity for dental fear treatment among their patients before considering facilitating in-person CBT.

#### Benefits.

Although participants generally opposed the in-chair option, some identified benefits of in-person treatment. A *relative advantage* highlighted is CBT’s potential to replace or reduce unnecessary medication (taken for anxiety). Some dentists also thought the in-chair treatment could be more feasible at certain times of the day. A few participants expressed that this intervention, if successful, would pay off in the longer run, reducing inconveniences imposed, and time wasted, by fearful patients. An aspect of *compatibility* emphasized across the focus groups/interviews is that an in-chair session may be the most effective option. Despite setbacks of using office space, some participants emphasized the importance of an authentic environment, relative to simulated or telehealth alternatives, to produce desired effects from treatment. One dentist commented, “I understand the importance of sitting in the chair and going through the motion and desensitization.”

### Step 2: Remote one-on-one telehealth

The second intensive treatment option is the remote one-on-one telehealth session, which would occur after the patient completes the app. This option allows patients to meet for one hour with a mental health provider over Zoom. Like the in-person option, the telehealth session also uses an exposure and other CBT approaches and is individualized to the patient’s fears.

#### Challenges.

Overall, focus group/interview participants were more receptive to this option compared with the in-person option. Challenges regarding the telehealth session were coded as, i*ncompatibility*, c*omplexity*, *lack of trialability*, and *lack of observability*. Some participants worried that a telehealth option may be less effective than in-person; dentists postulated that getting these patients into the actual dental chair is a necessary component of overcoming fear. Another aspect of *incompatibility* was dentists’ prediction that getting patients to show up for virtual appointments would be difficult. They suggested the accountability patients feel to show up is reserved for physical spaces/in-person appointments, and no-shows for telehealth would be frequent. One dentist commented that whereas the telehealth option seems more realistic than the in-chair option, they still don’t feel the need for any intervention — “I can usually manage most of my patients’ anxieties and fears with my present toolbox.” Some participants also voiced concern over the *complexity* of a telehealth intervention. One participant noted that it may be necessary for staff to act as a middle step to explain the intervention correctly and make patients comfortable with “a stranger who is not a dentist in a [virtual] dental office.” This added step for dental staff would create difficulty in treatment administration, burdening the practice.

Participants also highlighted the current *lack of trialability* and *lack of observability.* To integrate referral to a telehealth treatment into practice, participants expressed a need for data indicating treatment efficacy. They also requested the option to attend a mock session, allowing them to witness and evaluate the treatment process. In addition, some dentists stated that they would like to know how many patients would use this service and what types of patients would be most interested. One dentist noted that younger generations value mental health more than previous generations, so younger patients may be more inclined to receive treatment; others agreed.

#### Benefits.

Most participants found the telehealth treatment option more practical, financially feasible, and accessible than the in-person option. They felt that telehealth sessions would be easier to organize and place less burden on the dentist. A *relative advantage* that came up in multiple focus groups/interviews was the potential to reduce medications prescribed to manage patient anxiety. In addition, participants saw this option as an economic advantage. Since the telehealth sessions are done remotely, dentists wouldn’t have to worry about patients occupying dental chairs during office hours. An important aspect of c*ompatibility* emphasized was the convenience of telehealth treatment; participants agreed that this option would be more convenient, comfortable, and less stressful for their fearful patients. One dentist highlighted the advantage of being able to do the session from the comfort of one’s own home “as opposed to having to schlep into the dental operatory and do it there.” Participants also felt their patients would be more receptive to this option than an in-person one.

### Step 2: Virtual reality

The third option for dental fear intervention involves private practices purchasing a virtual reality (VR) headset with custom dental fear treatment software. After completing the app, patients who still have moderate-to-severe dental fear would be scheduled for a one-hour VR session at the office (when an empty chair is available).

#### Challenges.

Several challenges were similar to those proposed regarding the in-chair sessions. Although the avenues for intervention are distinct, both require office space. A noteworthy *relative disadvantage* of using VR was the time and cost inconvenience. Some participants commented that the telehealth session, which can be done from a patient’s home, is more feasible than the VR, which requires coming into the office and using a dental chair. Participants emphasized the financial disadvantages of using VR in their practices due to the cost of using office space and the cost of the headset itself. One participant voiced that it would be unwise for a dentist to buy the VR headset and allow office space to be used unless they were confident there would be a financial return. Regarding *incompatibility,* multiple participants speculated that if they were going to cede office space, they would prefer to use it for the one-on-one session as opposed to the VR. Dentists expressed that the human connection from therapy may be missing with virtual reality and preferred models that involve direct patient care by a therapist. Additionally, as proposed with the in-chair option, participants were wary of time constraints on their patients’ end. Factors related to *lack of observability* were also prevalent in the discussion of VR. Participants noted that they would need to see evidence of success rates before considering purchasing anything. Participants also reiterated needing to know the percentage of patients who would use the VR treatment method. Participants expressed interest in understanding the VR treatment itself and getting a sense of how it might appear to patients. One dentist commented that it’s important to know what the procedure would be like and to have “the script” before deciding whether to implement it.

#### Benefits.

Despite challenges, several dentists supported the use of VR headsets, identifying factors related to *relative advantage* and *compatibility*. Participants discussed the potential financial advantages of VR. One participant mentioned that the cost of the VR headset is quite reasonable, especially compared with the other treatment options; the group felt this would be more financially feasible than paying for a therapist. Additionally, some participants noted that the treatment could pay off in the long run. One participant commented that if the success rates were high, it would certainly be worth the purchase. In addition to advantages on the practice’s end, participants also described the benefits of virtual reality from a patient’s standpoint. Aspects of this *compatibility* involve the popularity of VR technology and similar tech innovations, particularly among younger generations. Participants tended to agree that the VR intervention seemed interesting and would like to learn more about the software.

## Discussion

This study was conducted to probe dentists’ attitudes toward three different models of dental fear treatment, all of which leverage technology to improve disseminability of CBT-DF, and each of which includes a dental practices’ participation to varying degrees. Consistent with current research [26,27], most dentists in our focus groups/interviews agreed that dental fear is an issue within their patient populations with observable negative effects on their patients’ oral health and their practices’ functioning. Dentists described experiences of fear leading to missed appointments or forcing them to stop work mid-procedure, both of which they felt were wastes of time, that sometimes had financial repercussions. The consensus that dental fear is an unresolved issue for practices facilitated some participant buy-in regarding the need to consider adoption of an intervention modality.

Results suggest that dentists are generally predisposed to the trialability of anxiety treatments for dental fear as summarized across the Rogerian themes (*relative advantage, compatibility, complexity, trialability,* and *observability*). Only a small minority of participants expressed the common misconceptions that fear is not an issue in their practices or that they can always identify and manage dental fear among their patients [10]. The development and adoption of behavioral health interventions that leverage technology (i.e., mobile apps, VR, telehealth) are novel interdisciplinary approaches that has recently served to increase collaborative care across different branches of medicine [25,28]. The growth of evidence-based, technology-delivered CBT is promising, particularly in fast-paced health settings [29,30]. Despite openness to such innovations, dentists expressed apprehension regarding feasibility.

Indeed, we found that most of the disadvantages on the dentist’s end were a result of using a dental chair (*relative disadvantage; in-chair one-on-one)*, which was eliminated with the telehealth option. On the patient’s end (from a dentist’s perspective), the issue of time (commuting to a dentist’s office) was a consistent barrier for both in-person options (in-chair and VR), again making telehealth a preferred method (*incompatibility; VR and in-chair one-on-one)*. Study results suggest the remotely administered telehealth option, with dental practices serving as referral partners, will be the most disseminable (*relative advantage and compatibility; telehealth one-on-one)*. However, it is important to keep in mind the two main disadvantages of this option proposed by participating dentists: potential decrease in patient accountability and treatment effectiveness (*incompatibility; telehealth one-on-one)*. Overall, these seemed like relatively minor concerns given the vast increase in accessibility and disseminability made possible by this approach. Patient accountability is a threat for both in-person and virtual options. Furthermore, rather than negatively impact attendance, as participants proposed, it is possible that the flexibility of telehealth leads to improved attendance (as suggested by recent research; [31]). Although an in-person exposure may more readily mimic a real dental session (i.e., using an actual dental chair, sights, and odors associated with dental procedures), telehealth exposures include other efficacious methods (e.g., imaginal techniques) and can be tailored to an individual patient’s dental fear via the use of precise sounds, images, and situational enactments. Although empirical inquiry in this domain is growing, more research on remotely administered exposure-based therapy for specific phobias is needed to (a) standardize effective protocols and then (b) test their efficacy relative to in-vivo.

Finally, the app itself (the first step of the intervention) was perceived by dentists as easy to implement, likely to promote engagement, and a familiar technology for most patients (*relative advantage, compatibility, and usability; app)*. Participants were interested in using the app as a screening instrument which could influence the dentists’ approach and reduce problematic interactions (*relative advantage; app)*. Universal fear assessment is considered best practice to appropriately tailor dental care for anxious patients [32].

Numerous studies exist on the efficacy of CBT-based interventions for dental fear [7,30,33], telehealth-adapted CBT treatments [34], and ways technology can be employed to facilitate mental health treatments across medical subspecialties [35]. This study furthers these lines of inquiry, considering dentists’ perspectives and opinions of different models of technology-facilitated psychological intervention from an implementation science perspective. Perceptions of the “characteristics of innovations” can determine how slowly or quickly they diffuse within a given system [24]. This qualitative research provides a lens through which to consider barriers to dissemination from a private practice standpoint. Despite the perceived advantages of in-person interventions, dentists found telehealth services more feasible for dentists and patients alike and believed other dentists were more likely to endorse them. Proposed considerations regarding practicality, workflow, chair time, and human connection may inform the development and adaptation of future models of psychological intervention for dental fear.

This study has several limitations. First, the sample size of this study is small (*N* = 13) and therefore not generalizable to all dentists. This is an exploratory study; consequently, we interviewed until arriving at thematic saturation. Future research is needed to replicate this research with a larger or more diverse sample. In addition, our sample is self-selected; therefore, it’s possible that the people who chose to participate already had an interest in the psychological well-being of their patients. With this in mind, it’s important to take into account the possibility of bias within this sample. Furthermore, although we could not tie specific quotes to dentists’ demographic information, and the sample size precludes making any generalizations of this nature, dentists’ age and gender may influence receptivity to behavioral health treatments [36]. This is a topic that warrants future research. Second, there was a miscommunication across several discussions regarding the financial repercussions of specific interventions. During multiple focus groups/interviews, participants seemed either under the impression that they would be responsible for paying the mental health provider who provided this treatment or were unclear about how the billing process would work (e.g., if this were something insurance would cover). This was not clarified explicitly by the interviewer, which likely resulted in the perception that certain cost barriers were greater than they would be. Although this misconception may have influenced the overall tenor of participant responses, its impact should be minimal given that all quotes related specifically to dental practices paying the mental health provider were excluded. Additionally, while we feel that Rogers’ lens was an appropriate choice for data analysis, we found little reference to factors indicative of trialability across discussions. The exception to this came up in a discussion of wanting to observe a “mock” session (*trialability; telehealth one-on-one)*.

This research sheds some light on gaps in the field, namely, the disconnect between dentistry and mental health. We found that lack of *observability* came up frequently as a barrier to implementing any of these interventions within a practice. There was a general lack of awareness regarding the well-developed evidence base for CBT treatments for mental health difficulties generally and for dental fear specifically. This highlights the need for proper dissemination (i.e., marketing and education), which in turn has the potential to increase engagement. Social media has been shown as an effective way to spread information and awareness to the general population [37]. This gap also highlights the need for the diffusion of evidence-based dental fear treatment information via dental student education, conferences, and dental associations. It is recommended that future research explore the most effective means of communicating information and evidence-based approaches regarding dental fear among providers so that practice community can stay up to date.

This study highlights the accessibility of mobile-based applications and telehealth interventions for treating dental fear when compared with in-person options. These focus groups/interviews provide insight into the necessity of interventions that will not disrupt dental practice workflow. Eventually, app-based and telehealth treatments have the potential to be disseminated throughout private practice dental practices.
